# Treatment algorithm in hormone-resistant prostate cancer: Practical guidelines

**DOI:** 10.4103/0970-1591.30271

**Published:** 2007

**Authors:** Makarand V. Khochikar

**Affiliations:** Department of Uro-oncology, Siddhi Vinayak Ganapati Cancer Hospital, Miraj, India

**Keywords:** Cancer-specific chemotherapy, castrate levels, hormone-resistant prostate cancer, psycho-oncology, secondary hormonal therapy, supportive care

## Abstract

Treatment of hormone-resistant prostate cancer can be a challenging situation. The first important step in treating this condition is to assess if one has achieved the castrate level or not. If the castrate levels are not achieved, attempt should be made to achieve so. If the castrate level is achieved, then androgen withdrawals may be of help. Supportive care, care of the clinical problems forms an integral part of the treatment. Cancer-specific chemotherapy is certainly an option in progressive disease.

The first important step in treating hormone-resistant prostate cancer (HRPC) is to find out if complete castrate levels are achieved or not. It has significant bearing on planning the further course of treatment. If the serum testosterone is at noncastrate level then further androgen suppression should be achieved.[1] If the castrate levels are achieved, then one could have options of either withdrawing antiandrogens or changing antiandrogens or trying intermittent androgen therapy or even trying secondary hormonal therapy. Continued androgen suppression with the same drugs or change of AA has been found to be effective in some patients.[2,3] Antiandrogen withdrawal has significant effect on the PSA decline – the first report came in 1993 as ‘Flutamide withdrawal syndrome’.[4] The overall response could be in the range of 15-33% lasting for more 3.5 + months to more than five months in various studies.[5–8]

Secondary hormonal therapy also has a significant role to play in HRPC. Its beneficial effect has been found to be in the range of 30-60% with drugs like ketocanazole and aminoglutethimide.[9–11] Use of diethylstilbestrol has shown a response rate of 20-40% in various studies.[12]

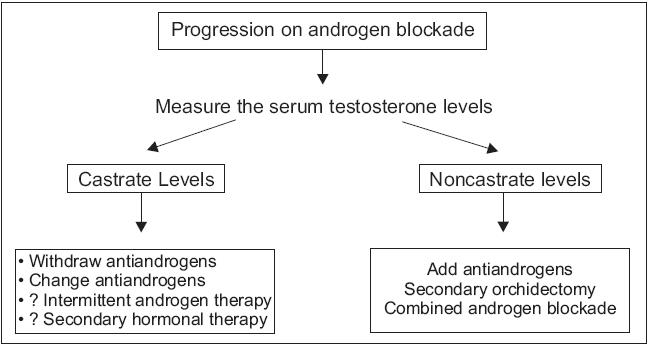



Secondary hormonal therapy may include DES, ketocanazole, prednisolone, finasteride, dutasteride, estramustine, aminoglutethimide, etc.

**Table:** 

Noncastrate levels	
Previous treatment	Plan of action

LH-RH analogues	Add antiandrogens[14–15]
LH-RH + antiandrogens	Change antiandrogens[16]
	Secondary orchidectomy
AA alone	Add LH-RH
	Secondary orchidectomy?
	Change AA
Bilateral orchidectomy	Add antiandrogens

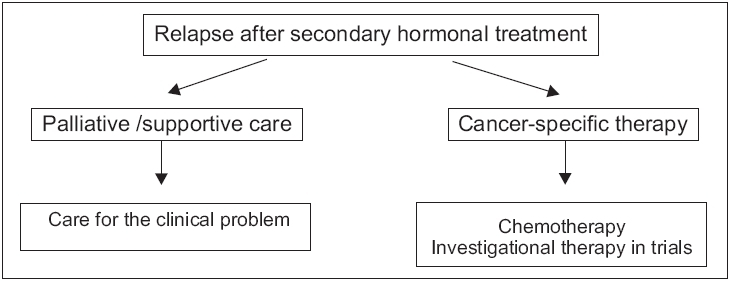



**Table:** 

Clinical problems and the care		
Clinical problem	Options	Comment

BOO	Indwelling catheter/SPC	High rate of incontinence
	Channel TURP	after TURP
	Prostatic stents	Blockage of stents
Hematuria	Bladder washout	Haemostatic RT quiet useful
	Haemostatic agents	
	Haemostatic RT	
Urinary incontinence	Indwelling catheter	Can be nuisance despite catheterization
Ureteric obstruction	Antegrade stenting	Most difficult problem is decision-making 'to
	PCN	treat or not to treat'.
	Steroids	Certainly not for dialysislndividual situations
	Honvon	may make decisions easier
	? leave alone	
Bony pains	NSAID	Use ‘WHO’ ladder for pain relief[17]
	Opiates	local RT effective
	Local RT	Side-effect profile of NSAIDs, opiates,
	Biphosphonates	strontium can be bothersome
	Strontium[18]	
	P-32	
	Steroids	
Pathological fractures	Stabilization	Fixation improves QOL and prevents inherent
	Postfixation-RT	complications of fractures[19]
Spinal cord compression	Steroids	Timely treatment essential for impending
	Local RT	neurodeficit
	Decompression	A
Anemia	RBC component therapy	Overenthusiastic treatment to be avoided[20]
	Blood transfusion	
	Erythropoietin	
	Bone marrow stimulants	
	Nutritional support	
Coagulopathy	Platelets, FFP?	Apart from the disease, look for any drugs
	Heparin	responsible for coagulation disorder
Lymphoedema	Steroids	Rarely useful
	Stockings	
Rectal obstruction/	? Colostomy	Rarely justified
rectovesical fistula	? ? ? Urinary diversion	
Psychological issues	Antidepressants	Psycho-oncology is an emerging branch. Such
	Mood elevators	professional help can be rewarding
	Counseling[21,22]	

AA - Antiandrogens, BOO - Bladder outflow obstruction, SPC - Suprapubic catheter, RT - Radiotherapy, FFP - Fresh frozen plasma

Secondary orchidectomy has a definite role to play if the castrate levels are not achieved. The response rate would be in the range of 5-70% depending on the prior hormone manipulation used and partly due to inconsistent use of the drugs.[13]
